# A Pilot Genome-Scale Profiling of DNA Methylation in Sporadic Pituitary Macroadenomas: Association with Tumor Invasion and Histopathological Subtype

**DOI:** 10.1371/journal.pone.0096178

**Published:** 2014-04-29

**Authors:** Chao Ling, Matthew Pease, Lingling Shi, Vasu Punj, Mark S. Shiroishi, Deborah Commins, Daniel J. Weisenberger, Kai Wang, Gabriel Zada

**Affiliations:** 1 Research Center of Basic Medical Sciences, Tianjin Medical University, Tianjin, China; 2 Zilkha Neurogenetic Institute, Keck School of Medicine, University of Southern California, Los Angeles, California, United States of America; 3 Department of Neurosurgery, Keck School of Medicine, University of Southern California, Los Angeles, California, United States of America; 4 USC Epigenome Center, Keck School of Medicine, University of Southern California, Los Angeles, California, United States of America; 5 Department of Radiology, Keck School of Medicine, University of Southern California, Los Angeles, California, United States of America; 6 Department of Pathology, Keck School of Medicine, University of Southern California, Los Angeles, California, United States of America; 7 Department of Psychiatry, Keck School of Medicine, University of Southern California, Los Angeles, California, United States of America; 8 Division of Bioinformatics, Department of Preventive Medicine, Keck School of Medicine, University of Southern California, Los Angeles, California, United States of America; 9 NCCC Bioinformatics Core and Division of Hematology, Keck School of Medicine, University of Southern California, Los Angeles, California, United States of America; CEA - Institut de Genomique, France

## Abstract

Pituitary adenomas (PAs) are neoplasms that may cause a variety of neurological and endocrine effects. Although known causal contributors include heredity, hormonal influence and somatic mutations, the pathophysiologic mechanisms driving tumorigenesis and invasion of sporadic PAs remain unknown. We hypothesized that alterations in DNA methylation are associated with PA invasion and histopathology subtype, and that genome-scale methylation analysis may complement current classification methods for sporadic PAs. Twenty-four surgically-resected sporadic PAs with varying histopathological subtypes were assigned dichotomized Knosp invasion scores and examined using genome-wide DNA methylation profiling and RNA sequencing. PA samples clustered into subgroups according to functional status. Compared with hormonally-active PAs, nonfunctional PAs exhibited global DNA hypermethylation (mean beta-value 0.47 versus 0.42, *P* = 0.005); the most significant site of differential DNA methylation was within the promoter region of the potassium voltage-gated channel *KCNAB2* (FDR = 5.11×10^−10^). Pathway analysis of promoter-associated CpGs showed that nonfunctional PAs are potentially associated with the ion-channel activity signal pathway. DNA hypermethylation tended to be negatively correlated with gene expression. DNA methylation analysis may be used to identify candidate genes involved in PA function and may potentially complement current standard immunostaining classification in sporadic PAs. DNA hypermethylation of *KCNAB2* and downstream ion-channel activity signal pathways may contribute to the endocrine-inactive status of nonfunctional PAs.

## Introduction

Pituitary adenomas (PAs) are typically benign monoclonal neoplasms with an overall prevalence of 16.7% (14.4% in autopsy studies and 22.5% in imaging studies) in the general population. The majority of PAs, however, are small and nonfunctional tumors, and only 0.16–0.20% of them are macroadenomas ≥ 10 mm in diameter [1], [2]. Although histologically benign, many PAs may cause significant morbidity due to their anatomical location, often resulting in tumor mass effect and neurological symptoms in addition to causing hormonal over-secretion or hypopituitarism. Invasion into surrounding anatomical structures (e.g. cavernous sinus invasion) remains a major barrier to achieving long-term tumor and disease control, especially in cases of functional PAs resulting in malignant endocrinopathies such as Cushing's disease or acromegaly [3]. With regard to the phenotype of invasion, PAs are often classified according to the Knosp grading system, in which one of five grades is assigned based on the degree of cavernous sinus invasion and relationship to the internal carotid artery [4].

Previous studies have shown that genetic mutations play an important role in the tumorigenesis of familial PAs, particularly those with heritable mutations in the multiple endocrine neoplasia I (*MEN1*) and aryl hydrocarbon receptor interacting protein (*AIP*) genes [5]. In sporadic PAs, however, it has been suggested that alterations in epigenetic regulation, and particularly DNA methylation, may play a particularly prominent role in PA tumorigenesis and invasion, likely via loss or reduced expression of tumor suppressor genes (TSGs) [6]. Many known TSGs have been shown to harbor C-phosphate-G (CpG) island hypermethylation in PAs, which is associated with and frequently results in silencing of TSGs. For example, DNA methylation within promoter and exon 1 regions of the cyclin-dependent kinase inhibitor (*p16/CDKN2A)* gene was found at a high frequency in NFAs [7], [8], and DNA methylation of the CpG island like cell-cycle regulatory genes of growth arrest and DNA-damage-inducible, gamma (*GADD45G*) [9] and apoptosis gene of rhomboid domain containing 3 (*RHBDD3*) have been associated with PA evolution [10]. Other studies have shown that tumor specific epigenetic silencing of cadherin 13, H-cadherin (*CDH13*) and cadherin 1, type1, E-cadherin (*CDH1*), alone or in combination, were involved in PA development and invasion [11], and CpG hypermethylation-mediated glutathione S-transferase pi gene (*GSTP1*) inactivation was a common finding in PAs potentially contributing to their invasive behavior [12]. Few prior studies, however, have utilized genome-scale approaches according to functional PA subtypes and phenotypical invasion status to identify candidate genes involved in these processes [13], [14].

Although several targeted genes have been associated with PA tumorigenesis and progression, primary whole genome-scale epigenetic alterations remain largely unknown, especially as they pertain to invasion and histopathological PA subtype classification. In the current study, we utilized genome-scale DNA methylation technology to identify DNA methylation alterations between invasive and noninvasive PAs subtypes, and across varying functional classes of PAs.

## Materials and Methods

### Patients and tissue specimens

This study was conducted according to the Helsinki human subject doctrine and was approved by the Institutional Review Boards of the Keck School of Medicine of the University of Southern California (Los Angeles, USA). Written informed consent was signed and obtained from 23 participants and one guardian on behalf of a minor enrolled for tissue specimen collection and subsequent analysis. Twenty-four patients with surgically-resected PAs from the University of Southern California (USC) Keck Hospital and Los Angeles County + USC Medical Center were included in this retrospective study.

Of the 24 PAs, six (25%) were functional and 18 (75%) were nonfunctional. More specifically, these included 17 (70.8%) nonfunctional adenomas, 5 (20.8%) somatotroph adenomas, 1 (4.2%) corticotroph adenoma, and 1 (4.2%) silent corticotroph adenoma. All PAs analyzed were macroadenomas ≥ 10 mm in diameter, and were diagnosed based on laboratory evaluation and Magnetic Resonance Imaging (MRI), with an average tumor diameter of 23.55 TR×19.36 CC mm (Table 1). Knosp invasion scoring on MRI was performed by a staff neuro-radiologist who was blinded to the genomics analysis. For the purposes of this study, PAs with Knosp scores of 0–1 were classified as noninvasive, and those with Knosp scores of 2–4 were classified as invasive. Equal numbers of invasive and noninvasive PAs (12 each) were selected.

**Table 1:** Patient and tumor characteristics.

Patient	ID	Age	Gender	Invasion	Knosp	Subtype	Size (TR×CC)	Ave_beta Value
1	d9986	66	M	0	0	Silent ACTH	NA	0.5
2	d0116	33	F	0	0	NFA	21.75×22.61	0.51
3	d0261	46	F	0	0	NFA	18.94×15.62	0.55
4	d0500	70	M	0	0	NFA	NA	0.53
5	d1055	38	M	0	0	GH	19.64×12.37	0.43
6	d1438	43	M	0	0	NFA	24.08×20.94	0.52
7	d0298	16	M	0	1	GH-LH	13.68×14.25	0.43
8	d8312	55	F	0	1	NFA	20.55×15.32	0.51
9	d0079	69	F	0	1	GH-TSH	28.93×16.85	0.47
10	d9827	92	M	0	1	NFA	15.94×8.69	0.55
11	d1426	49	F	0	1	GH	18.67××14.39	0.41
12	d0266	49	M	0	1	NFA	23.74×18.94	0.5
13	d0628	55	F	1	2	NFA	25.93×25.17	0.41
14	d0666	45	F	1	2	NFA	17.41×13.59	0.45
15	d8485	57	F	1	2	NFA	30.75×29.91	0.5
16	d8190	67	M	1	2	NFA	19.47×22.49	0.5
17	d8279	40	F	1	2	NFA	25.41 ×33.26	0.47
18	d9620	53	M	1	3	NFA	NA	0.48
19	d9988	31	F	1	3	NFA	28.50×23.18	0.54
20	d8277	43	M	1	4	GH-PRL	40.04×22.42	0.43
21	d8447	59	M	1	4	NFA	28.42××20.18	0.49
22	d1341	66	F	1	4	NFA	32.22×18.8	0.51
23	d1447	52	F	1	4	ACTH	16.60 ×17.94	0.5
24	d0413	60	F	1	4	NFA	NA	0.48

M: Male, F: Female; Age: Year unit; PRL: Prolactin; TR × CC: Transverse (mm) × Antero-posterior (mm). The average size of all PAs was 23.55 TR mm × 19.36 CC mm; NA: Not applicable. Invasion: “0” represents noninvasive PAs, “1” represents invasive PAs; Ave: Average; Knosp: Knosp grade of PAs.

Following transsphenoidal tumor resection, PA specimens were fresh-frozen in liquid nitrogen and stored at −80°C until used for DNA and RNA extraction. Genomic DNA purification was processed using DNeasy Blood & Tissue kit (Qiagen) according to the standard protocols of the manufacturer. Total RNA was purified using RNeasy Plus Universal Kits (Qiagen). Tissue samples were first cut into small pieces with no more than 50 mg on dry ice then moved promptly into 1.5 ml precooled tubes. Liquid nitrogen was then added into the tube, and tissue was grinded with a grinding rod before adding 0.9 ml of QIAzol Lysis Reagent. The tubes were vortexed for 60 seconds and left at room temperature for 5 minutes before following the standard RNeasy Plus protocol for RNA purification.

### Genome-scale DNA methylation array

One microgram of bisulphite converted DNA (using the Zymo EZ DNA Methylation kit, Zymo Research, Irvine, CA) per sample was used for genome-scale DNA methylation profiling, and was performed in the USC Epigenome Center using the Illumina Infinium HumanMethylation450 (HM450) Beadchip platform, which interrogates 482,421 DNA methylation sites and covers 99% of Reference Sequence of National Center for Biotechnology Information (NCBI-RefSeq) genes, with an average of 17 CpG sites per gene region distributed across the promoter, TSS1500 (1500 bp within transcription start site), TSS200 (200 bp within transcription start site), 5′UTR, 1stExon (the first exon), gene body, 3′UTR. It covers 96% of CpG islands, with additional coverage in island shores and the regions flanking them. The DNA methylation data was submitted to Gene Expression Omnibus (GEO) with access number of GSE54415. Beta values for all HM450 probes were calculated after background correction and normalization as previously described [15]. Probes (11,648) located on chromosomes X and Y were excluded. Probes targeting non-CpG sites (3,091) and probes targeting 65 known single nucleotide polymorphisms (SNPs) were also excluded. An additional set of 86,560 probes with common SNPs at the CpG site with a minor allele frequency (MAF) greater than 1% (identified using NCBI dbSNP builds 128), or with common SNPs within 10 bp from CpG site, or within 15 bp from the CpG site lying entirely within a repeat region (from RepeatMaster and Tandem Repeat Finder databases) were excluded. Finally, 591 probes with detection P-values >0.01 were removed. Following these filtering steps, a total of 383,718 probes were utilized for analyses (Figure A in File S1). We determined the distribution of each probe by CpG density (island, shore, shelf, etc) and by location (promoter, gene body, UTR, etc.) (Figure A and B in File S2). A secondary analysis of probes with beta values <0.1 across 77.8% (14/18 samples) of nonfunctional adenomas were found to be less variable and therefore could conceal the potential variance. These probes were excluded in order to investigate the underlying difference between invasive and non-invasive nonfunctional adenomas. Remaining subsets of 884 probes were selected for the in-depth analysis (Figure B in File S1). Finally, probes with Δbeta >± 0.3 were used in differentiating various PA classes based on phenotype.

**Figure 1:**
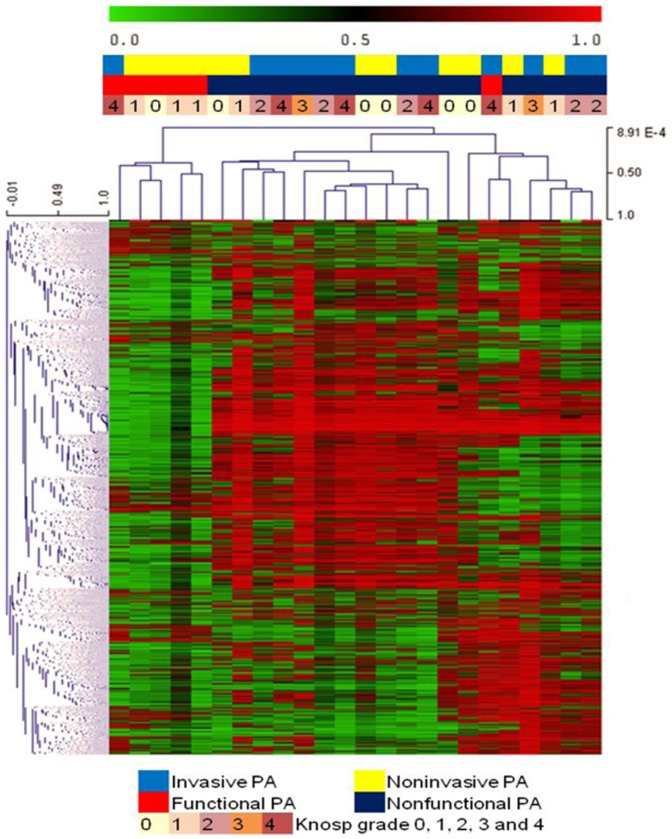
Hierarchical Clustering of DNA methylation in 24 PA cases. The HM450 probes (7,674) with the highest 2% of standard deviation across the set of 383,718 global HM450 probes were used.

Hierarchical Clustering (HCL) was performed using the Microarray Software Suite of MultiExperiment Viewer (MeV) V4.8 [16] using probes with the top 1%, 2% and 5% of standard deviation (SD) across the set of 383,718 “global” probes in 24 PA samples respectively. Two-way clustering was performed using Pearson's correlation and average linkage for both the gene tree and sample tree (Figure 1 with top 2% SD). Differential DNA methylation analyses were performed between: 1) Invasive versus noninvasive PAs (12 samples each), 2) FA subjects (n = 6) versus NFA subjects (n = 18), 3) Invasive NFAs (n = 10) versus noninvasive NFAs (n = 8), and 4) PAs and NFAs stratified according to the five different Knosp invasion grades (0–4). Two tailed t-test analyses were performed between FAs versus NFAs and noninvasion versus invasion PA. Kruskal-Wallis test was subsequently used to analyze DNA methylation differences among 18 NFA subjects with five different Knosp grades (Table 1). All of the analysis were executed in globe probes with the proportion of false significant genes did not exceed 0.01.

### MethyLight DNA methylation analysis

Validation of findings from DNA methylation profiling in *KCNAB2,* which spans the CpG site interrogated by the Illumina HM450 array at probe cg18192083, was performed using MethyLight real-time PCR assays as previously described [17]. One microgram of genomic DNA from each of the 24 PA samples and the CpG methyltransferase (M.*Sss*I) treated reference DNA sample was converted with bisulfite using the Zymo EZ DNA Methylation kit (Zymo Research, Irvine, CA), as specified by the manufacturer. A control reaction targeting *ALU* repetitive elements [18] was used to normalize the input bisulfite-DNA amounts. The real-time PCR reactions were obtained from Life Technologies, Carlsbad, CA. The *KCNAB2* MethyLight primer and probe sequences are: *forward: 5′-*GGT TTT TTT ATT TGG GTT ACG CG-3′; *reverse:* 5′-CGC AAA ACT AAA AAA CCT AAC GC-3′; *probe: 5*′*-*6FAM-CCG TAA AAT ATC GAA ACG TAA CC-MGBNFQ-3′, in which 6FAM is the fluorophore and MGBNFQ refers to a minor-groove binding non-fluorescent quencher. The *ALU* control reaction primer and probe sequences were previously described [18]. The data were reported as a Percent of Methylated Reference (PMR), calculated as: PMR  =  (((*KCNAB2/ALU*)_ sample_)/(*KCNAB2*/*ALU*)_ M.SssI_)) × 100.

Using HM450 manifest (downloaded from gene expression omnibus, GPL13534) 86,772 promoter-associated CpG probes were selected for the pathway analysis using gene set enrichment analysis (GSEA) software [19]. A total of 1,454 gene sets of Human Gene Ontology were exported from the molecular signatures database (MSigDB) version 3.1, and after filtering, 469 of the gene sets were either larger than 500 or smaller than 25 gene set size allowed. Additional statistical analysis was performed using R software (http://www.r-project.org/) and GraphPad Prism Software (GraphPad Software Inc.).

### Whole-genome gene expression sequencing

One microgram of total RNA from each of the 24 samples was assessed by Agilent Bioanalyzer to examine quality, and subsequently used for library preparation. Library quantization was performed by Kapa Biosystems qPCR assay. RNA sequencing was performed in the USC Epigenome Center using Illumina Hi-seq 2000 platform to produce 100 bp paired-end reads, by indexing four samples per lane. The Fastq files could be downloaded from Sequence Read Archive (SRA) with accession number SRP035646. Raw sequence data were first examined by FastQC as a quality control measure. We next used Tophat and Cuffdiff to examine genes with differential expression, based on the fold change of fragments of per kilo base of transcript sequence per millions base pairs (FPKM).

## Results

### Genome-wide DNA methylation in invasive and noninvasive PAs

**Figure 2:**
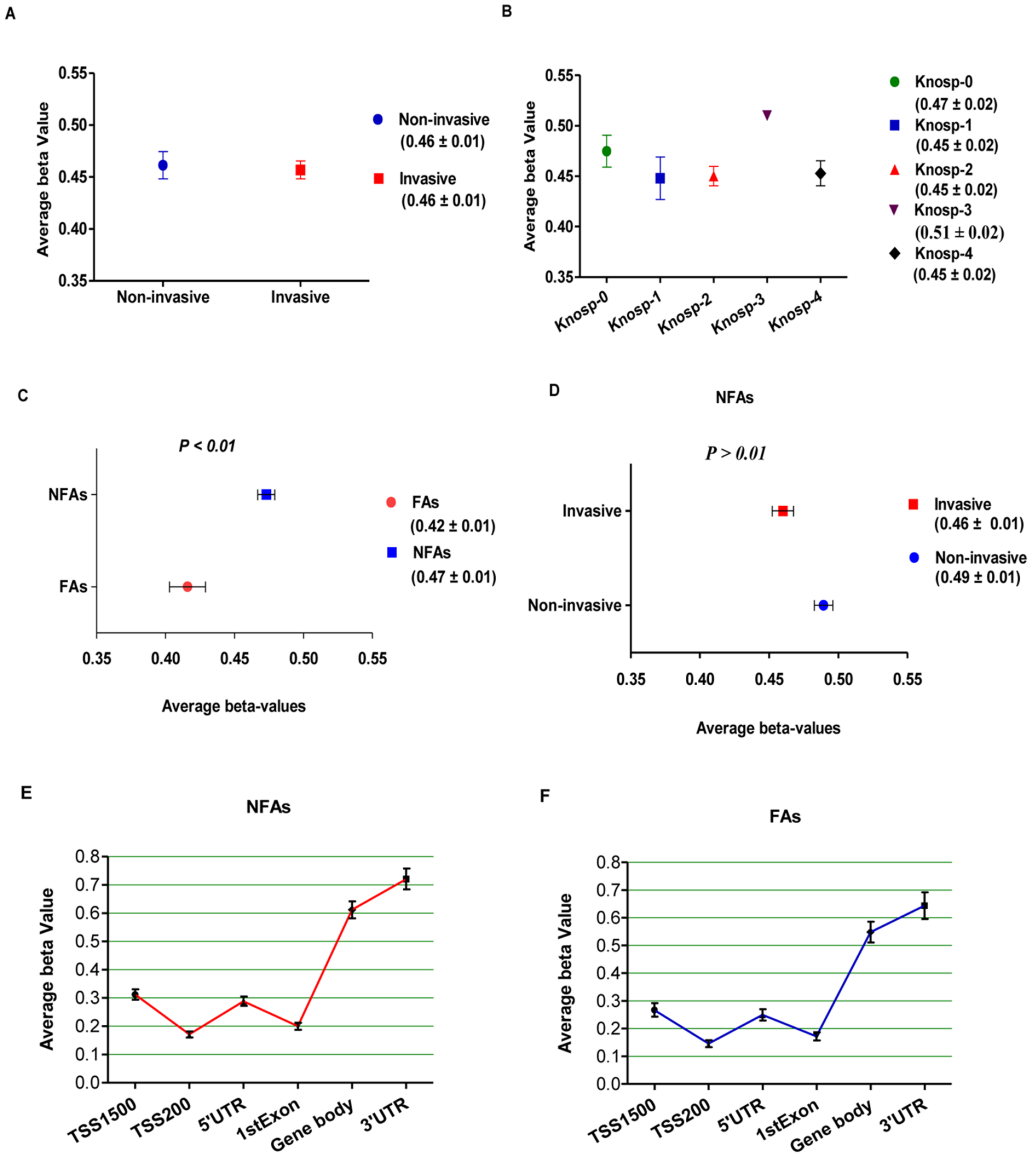
Global CpG DNA methylation profiles in PAs. A, Comparison of mean DNA methylation levels across 383,718 HM450 probes in noninvasive (Knosp grade 0, 1) and invasive (Knosp grade 2, 3, 4) PAs. B, Mean DNA methylation levels in PA cases across the five different Knosp grades (0–4) among the set of 383,718 HM450 probes. C, Comparison of mean DNA methylation levels across 383,718 HM450 probes in FAs and NFAs. P-value was calculated via simple t-test. D, Comparison of mean DNA methylation levels across 383,718 HM450 probes in only NFAs. P-value was calculated via simple t-test. All of the above DNA methylation beta values are shown as mean ± SEM in different groups. E and F, DNA methylation levels in gene regions (TSS1500, TSS200, 5′UTR, 1stExon, gene body and 3′UTR) for NFAs (panel E) and FAs (Panel F). TSS200: 200 bp within transcription start site; TSS1500: 1500 bp within transcription start site; UTR: untranslated region; 1stExon: the first exon of gene.

In this pilot study, twenty-four surgically-resected sporadic PAs with varying histopathological subtypes and Knosp invasion scores were characterized to determine if genome-wide and gene-specific methylation levels can serve as biomarkers of PA subtype and invasion grading. We calculated the mean DNA methylation beta value for the entire filtered probe set (383,718 probes) as a measure for global DNA methylation in noninvasive and invasive PAs (Figure 2A), across different tumor grades by Knosp categorization (Figure 2B), and in FAs and NFAs (Figure 2C). Based on these calculations, no significant differences in global DNA methylation levels were observed between the invasive and noninvasive groups (Figure 2A). Furthermore, Kruskal-Wallis test analysis among PAs with five different Knosp grades using the global filtered probe set also did not show significant DNA methylation differences as a function of tumor grade (Figure 2B), even though we relaxed the threshold to FDR <0.05. Within the set of 18 NFA subjects, similar (nonsignificant) findings between invasive and noninvasive PAs were also seen (Figure 2D). These data suggest that global DNA methylation profiles may not independently predict clinically significant differences in the invasive phenotype of PAs.

We further refined the probe set for unsupervised analyses to remove non-variably methylated probes. After filtering, 884 probes were selected, of which 355 were enhancer-associated, 20 were promoter-associated, and 4 were both promoter- and enhancer-associated based on the Illumina HM450 manifest for probes (Figure B in File S1). We identified 34 CpGs (localized to 17 genes) that were independently associated with enhancers and were hypomethylated in invasive NFAs compared to noninvasive NFAs (FDR <0.01) (Table S1). These differentially-methylated genes included the fms-related tyrosine kinase 1 (*FLT1*) and slit homolog 3 (*SLIT3*), which are two important genes known to be associated with cell motility and invasion. Although we attempted to validate methylation status in these two genes using MethyLight, we were not able to because the probes lie in CpG-depleted regions of the genes. No DNA methylation differences in promoter regions were noted between invasive and noninvasive NFAs.

### Nonfunctional PAs are globally hypermethylated compared to somatotroph PAs

The group of 24 PA samples independently clustered into two subgroups of nonfunctional and functional (all somatotroph) PAs following HCL with top 2% of SD (Figure 1). All somatotroph adenomas were exclusively clustered from NFAs (and one additional functional corticotroph adenoma) (Figure 1), and further clustering of the top 1% (File S3) and 5% (File S4) of SD validated this finding. The mean global beta values from FA (six subjects) and NFA (18 subjects) tumors showed that NFAs were significantly DNA hypermethylated compared to FAs (Figure 2C). However, NFAs and FAs showed similar DNA methylation trends from gene promoters through gene bodies and UTR regions (Figure 2E and F). These findings suggest that DNA methylation analysis may provide insight into hormonal activity in functional versus nonfunctional PAs, and possibly complement current PA histopathological classification systems.

**Figure 3:**
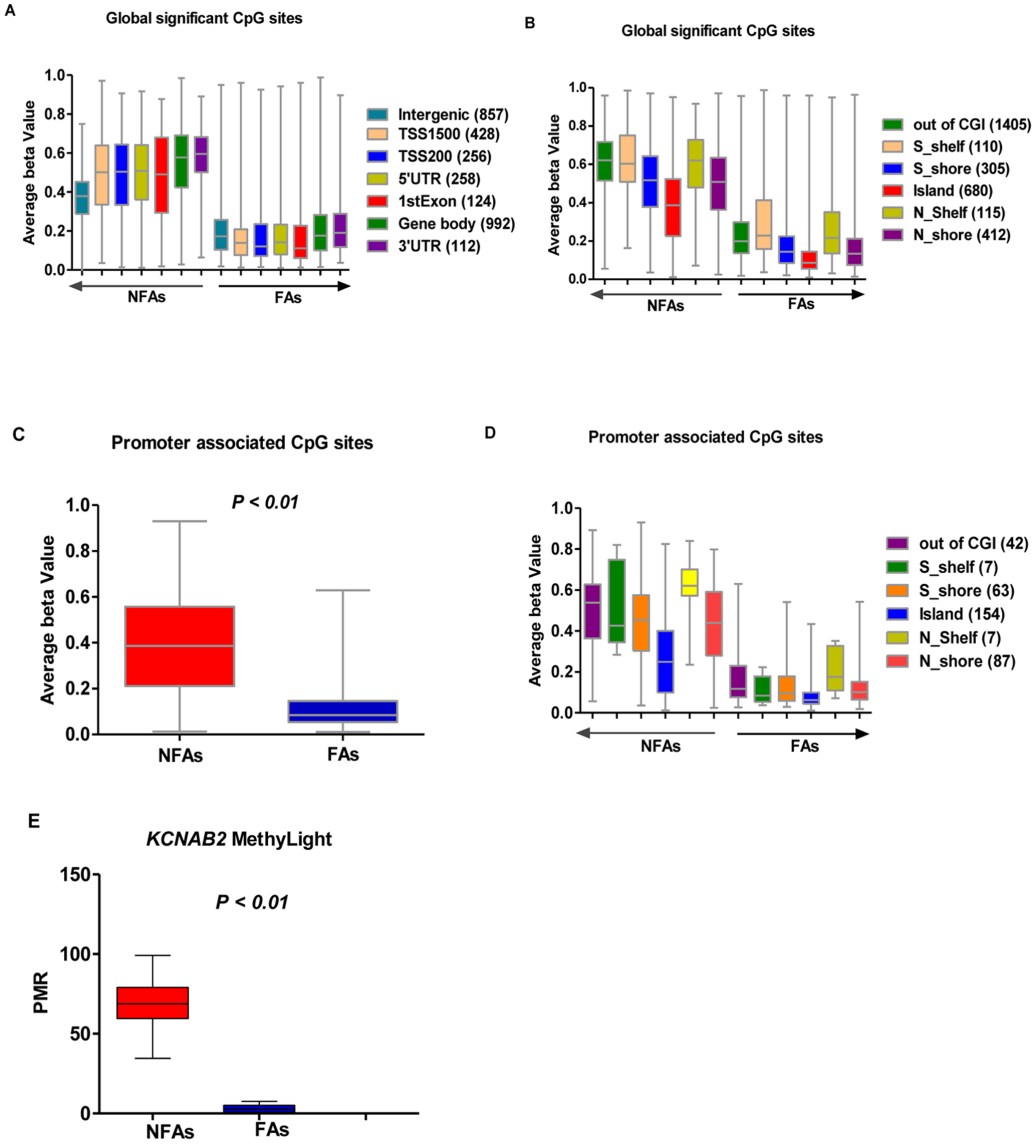
Characteristics of significantly methylated CpG sites in PA cases. Whiskers were used to represent the min to max beta values. A-D, Panel of 3,072 CpG sites located in Ref-Seq genes were interrogated for their DNA methylation in NFAs compared to FAs for all probes and stratified by location relative to each gene region and CpG density. A, The distribution of differentially methylated CpGs across different gene related regions. B, Stratified by CpG density. CpG islands (CGI), CpG island shores (0–2 kb from island edge, N and S indicate the upstream and downstream of the island, respectively), CpG island shelves (0–2 kb from shore edge, N and S indicate the upstream and downstream of the island, respectively) and non-CpG island probes. C and D, Relative distribution and count of the promoter associated CpGs (total 360), which were singled out from the global significant CpG sites (total 3027). E, *KCNAB2* (cg18192083) MethyLight assay in NFA and FA, which was consistent with the genome-wide DNA methylation analysis.

In order to interrogate which subsets of genes were involved in distinguishing specialization of nonfunctional PAs, we filtered the global panel of 383,718 HM450 probes to include those targeted in RefSeq genes for a more thorough investigation. Using a two-tailed t-test, we identified 3,027 CpG sites with significant DNA methylation differences between NFAs and FAs (FDR <0.01). In order to display the methylation status of the significant CpG sites, we graphed DNA methylation levels of these probes stratified by location relative to gene region (Figure 3A) and as a function of CpG density (Figure 3B). We further restricted the set of 3,072 probes to 360 CpG sites associated with promoter regions, and found substantial DNA hypomethylation in FA cases compared to NFA cases (simple t-test, P<0.01) (Figure 3C). Furthermore, CpG sites were stratified by density (Figure 3D) as well. The most significant gene with regard to differential DNA methylation was *KCNAB2*, a member of the potassium voltage-gated channel, shaker-related subfamily (FDR = 5.11×10^−10^), which was hypermethylated in the NFA group. This result was subsequently validated using MethyLight real-time PCR assays (Figure 3E). Furthermore, we found that among the top 30 most significantly differentiated genes, 4 (*NME9*, *PSEN2, MDN1* and *PCSK6)* were hypomethylated in NFAs compared to FAs (Table 2). Interestingly, we also corroborated the recent finding discovered using Illumina Infinium Methylation 27K Arrays that the transcription factor AP-2 epsilon (*TFAP2E*) and 13 other genes (Table S2) were hypermethylated in NFAs [13] (Table 2). Epigenetic alteration of the *TFAP2E* gene has also been commonly reported in selected human cancers, including prostate cancer and colorectal carcinoma [20], [21]. In addition, Wnt signaling plays an important role in adenoma development and tumorigenesis, including PAs [22]. Whether hypermethylation of *TFAP2E* could down-regulate Wnt signaling, however, was still unknown. In order to further understand the detailed DNA methylation status of *KCNAB2*, we performed hierarchical clustering of beta values from *KCNAB2*-targeted probes on the HM450 array using the top 50% highest standard deviation. In nonfunctional PAs, *KCNAB2* was generally hypermethylated across the promoter region (TSS1500 and TSS200), 5′UTR, the first exon, and gene body. This was substantially different from the coverage profile observed in functional PAs (File S5). Since *KCNAB2* is expressed abundantly in the nervous system and T lymphocytes, and appears to play an important role in K^+^ channel activation [23] and subsequent hormone secretion [24], we further extended our investigation of this pathway.

**Table 2:** Top 30 significant genes with differential DNA methylation between NFAs and FAs.

Gene_Symbol	Probe_CpG^a^	Target	NFA-M	SD^b^	FA-M	SD	NFA-FA^c^	p-value	FDR^d^
*KCNAB2*	cg18192083*	TSS200^e^	0.77	0.11	0.06	0.02	0.71	1.33×10^−15^	5.11×10^−10^
*CIITA*	cg09015246	1stExon	0.76	0.13	0.13	0.04	0.63	3.44×10^−14^	6.60×10^−9^
*SPAG17*	cg12435551	Body	0.77	0.15	0.06	0.05	0.7	6.15×10^−14^	7.87×10^−9^
*TFAP2E*	cg22851880	TSS1500^f^	0.79	0.14	0.13	0.05	0.66	2.02×10^−13^	1.94×10^−8^
*KCNAB2*	cg03400374*	TSS200	0.79	0.1	0.08	0.06	0.71	7.27×10^−13^	4.65×10^−8^
*SOCS1*	cg10784813*	3′UTR	0.7	0.15	0.16	0.06	0.53	1.88×10 ^−11^	8.03×10^−7^
*KCNAB2*	cg12604162*	TSS1500	0.74	0.11	0.13	0.06	0.62	2.43×10^−11^	8.49×10^−7^
*NME9*	cg05652381	5′UTR	0.23	0.2	0.86	0.05	−0.63	3.46×10^−11^	1.11×10^−6^
*SNORD115-48*	cg27513586	TSS1500	0.52	0.11	0.15	0.03	0.37	4.15×10^−11^	1.14×10^−6^
*TFAP2E*	cg13558810	TSS1500	0.7	0.17	0.15	0.04	0.55	4.11×10^−11^	1.21×10^−6^
*SNTG1*	cg21177502	5′UTR	0.75	0.16	0.25	0.05	0.5	1.56×10^−10^	2.85×10^−6^
*PSEN2*	cg05134019	5′UTR	0.23	0.17	0.81	0.07	−0.58	1.21×10^−10^	2.91×10^−6^
*TFAP2E*	cg26372517	5′UTR	0.71	0.2	0.08	0.03	0.64	1.52 ×10^−10^	2.92×10^−6^
*FAM26D*	cg12354014	5′UTR	0.57	0.14	0.13	0.05	0.44	1.30×10^−10^	2.94×10^−6^
*NEAT1*	cg14758218	Body	0.68	0.2	0.05	0.01	0.64	1.70×10^−10^	2.97×10^−6^
*TNFRSF11A*	cg16174779	Body	0.74	0.18	0.16	0.07	0.58	1.49×10^−10^	3.02×10^−6^
*UNC5A*	cg03320873	Body	0.75	0.21	0.16	0.05	0.59	2.00×10^−10^	3.34×10^−6^
*MERTK*	cg06682024	Body	0.61	0.17	0.09	0.03	0.52	2.47×10^−10^	3.80×10^−6^
*CAPN2*	cg06756211*	Body	0.7	0.23	0.04	0.05	0.66	2.44×10^−10^	3.90×10^−6^
*MDN1*	cg27302675	Body	0.24	0.22	0.88	0.06	−0.64	2.77×10^−10^	3.94×10^−6^
*PIK3R1*	cg24797508*	Body	0.63	0.18	0.09	0.06	0.54	2.68×10^−10^	3.95×10^−6^
*PCSK6*	cg10845124	Body	0.37	0.16	0.86	0.02	−0.49	3.33×10^−10^	4.26×10^−6^
*KCNQ4*	cg15867428	Body	0.56	0.17	0.08	0.05	0.48	4.12×10^−10^	4.39×10^−6^
*PHLDB1*	cg08473858	TSS1500	0.66	0.18	0.15	0.04	0.51	4.05×10^−10^	4.57×10^−6^
*SLC7A5*	cg08617020	Body	0.73	0.14	0.32	0.04	0.41	3.72×10^−10^	4.61×10^−6^
*LGALS3*	cg02183170	TSS200	0.74	0.18	0.14	0.08	0.6	4.02×10^−10^	4.68×10^−6^
*SEMA5B*	cg04830808	5′UTR	0.52	0.15	0.09	0.04	0.43	3.99×10^−10^	4.78×10^−6^
*AEBP1*	cg08739576	1stExon	0.22	0.06	0.06	0.01	0.16	5.12×10^−10^	4.91×10^−6^

a Probe_CpG: the Illumina HM450 probe ID.

b SD: standard deviation.

c NFA-FA: the mean beta value subtractive difference between NFA tumors and FA tumors.

d FDR: False Discovery Rate.

e TSS200: 200 bp within transcription start site.

f TSS1500: 1500 bp within the transcription start site.

* Promoter Associated.

Link to the whole information of the Probe_CpG: http://www.ncbi.nlm.nih.gov/geo/query/acc.cgi?acc=GPL13534.

### Methylation of ion channel activity signal pathway genes may be associated with PA functional status

Because gene promoter DNA hypermethylation is known to play an important role in tumorigenesis [25] and contributes to loss of gene function and silencing [26], we selected promoter-associated probes according to the Illumina HM450 manifest for further pathway analyses. We found that four gene sets were enriched (GSEA reports the gene sets with FDR <0.25) in NFAs, and no such gene sets were detected in FAs (Table S3, File S6). The most significant gene set was related to ion-channel activity signaling. Twenty core enrichment genes are shown in Table S4 and the pathway heat map is shown in File S7. The genes enriched in the pathway encoded K^+^, Cl^−^ and Ca^2+^ ion-channels, which are known to play important roles in hormone secretion [27], [28]. In order to determine whether there were any pivotal genes driving these pathways, we performed a leading edge analysis of the four significant gene sets. Two core genes, *KCNMB4* (potassium large conductance calcium-activated channel, subfamily M, beta member 4) and *CACNA1C* (calcium channel, voltage-dependent, L type, alpha 1C subunit) overlapped in the significant gene sets, and the ion-channel activity signal gene set highly overlapped with the substrate-specific channel activity signal gene set (Figure A and B in File S8). These data suggest that ion-channel pathways may play an important role in the process of hormonal secretion in functional PAs. As such, identified hypermethylation pathway genes, and in particular *KCNAB2,* may provide new insight to the epigenetic differentiation of FAs and NFAs.

### Association of DNA methylation with gene expression in sporadic PAs

Gene expression can be modulated in multiple ways, from transcription to post-translational modification. Epigenetic modification may affect gene expression levels at multiple steps, including DNA methylation, histone modification, chromatin remodeling and transcription factor interaction. To examine whether DNA methylation was associated with gene expression in PAs, we performed differential gene expression analysis among the 24 subjects using RNA-Seq.

**Figure 4:**
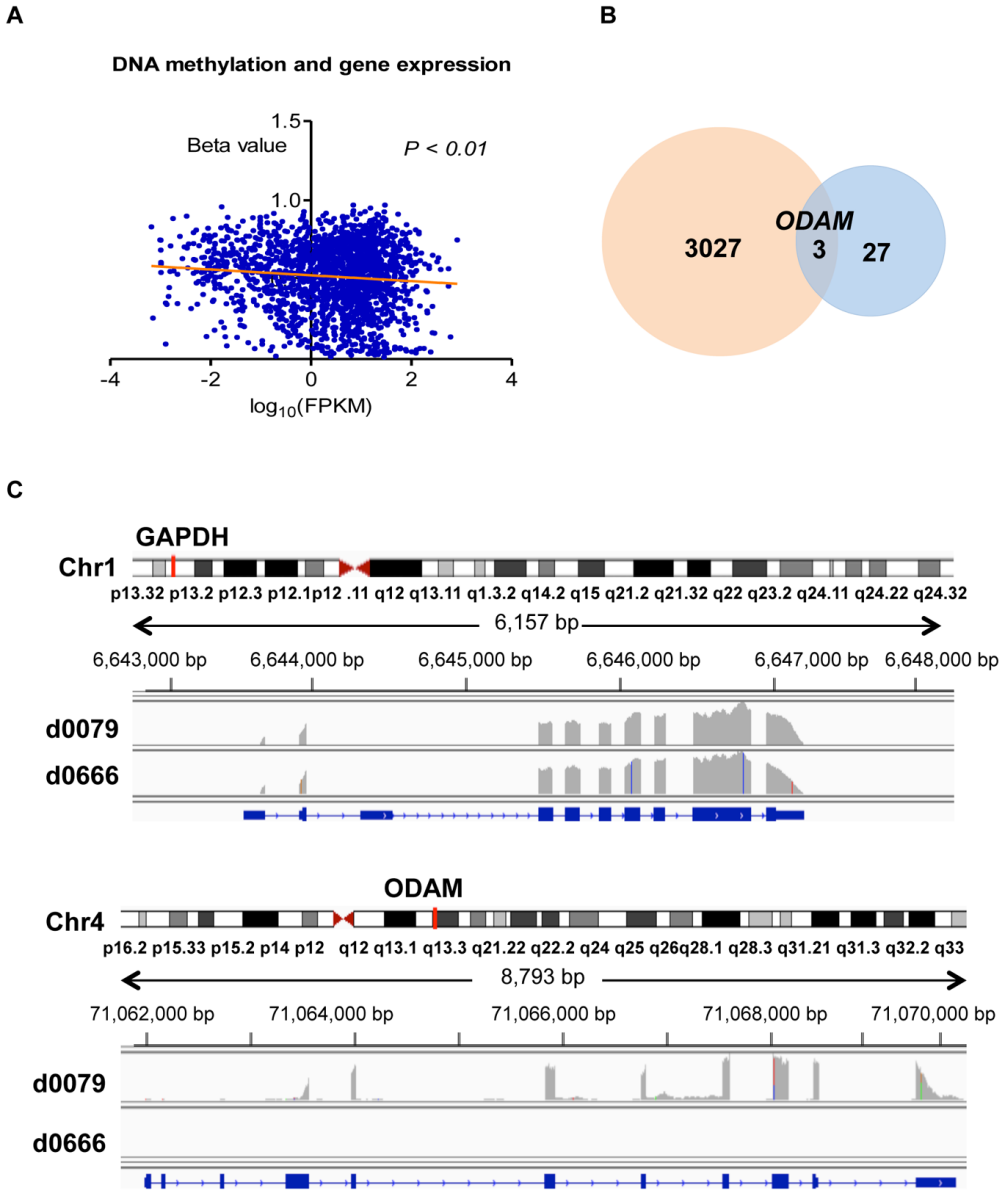
Gene expression. A, Linear regression analysis showed negative trend of DNA methylation and gene expression. B, Three overlapped significant genes of DNA methylation and gene expression when compared NFAs to FAs. C, gene *ODAM* expression in one FA subject of d0079 which secreted hormones of GH and TSH, *GAPDH* gene expression was used as control.

Comparison between NFAs and FAs showed that gene expression was not found to be significantly different (FDR<0.01) between the two groups. However, when relaxing the threshold to claim statistical significance, 27 genes (FDR<0.05) displayed a potentially different expression profile (Table 3). Based on the globally significant methylated CpG sites, we selected the relative hypermethylation genes in NFAs and investigated their gene expression patterns. We found that the trend of gene expression was negatively associated with DNA methylation (Figure 4A). When matching those 27 genes with significant genes identified by DNA methylation analysis, three genes, including ribosomal protein S6 kinase, 90 kDa, polypeptide 2 (*RPS6KA2),* retinol dehydrogenase 10 (*RKH10*), and odontogenic ameloblast associated protein (*ODAM*) were identified with altered methylation CpG sites in the gene body (Figure 4B). We found that genes *RDH10* and *ODAM,* which were hypomethylated in the CpG island and enhancer region, showed increased expression in FAs compared to NFAs. However, the *RPS6KA2* gene, which was hypermethylated in a CpG site in the gene body, also showed increased expression in FAs compared to NFAs. Finally, we found that *ODAM* was expressed in 16.7% (1/6) of FAs but showed no expression in NFAs (0/18) (Figure 4C). These results indicate that the function of DNA methylation seems to vary with context, and the relationship between DNA methylation and transcription is more complex than previously expected [29].

**Table 3:** List of genes with most significant differences in expression between NFAs and FAs.

Gene	Locus	FA-FPKM^a^	NFA-FPKM	p_value	FDR
*ASAP2*	chr2:9346893–9545812	41.37	6.20	5.0×10 ^−5^	0.03
*ATP1B2*	chr17:7554253–7561089	9.37	99.48	5.0×10 ^−5^	0.03
*CDK18*	chr1:205473683–205501921	3.95	61.97	5.0×10 ^−5^	0.03
*CNPY1*	chr7:155293952–155326539	0	0.44	5.0×10 ^−5^	0.03
*COL9A2*	chr1:40766162–40782939	91.12	5.10	5.0×10 ^−5^	0.03
*CSH1*	chr17:61972267–61974021	0.57	0	5.0×10 ^−5^	0.03
*CSHL1*	chr17:61986964–61988618	1.09	0	5.0×10 ^−5^	0.03
*FAM138E*	chr15:102495087–102496558	0	0.49	5.0×10 ^−5^	0.03
*FDCSP*	chr4:71091787–71100968	2.05	0	5.0×10 ^−5^	0.03
*GNB3*	chr12:6949374–6956557	25.85	287.08	5.0×10 ^−5^	0.03
*ITPR2*	chr12:26488284–26986131	2.88	18.21	5.0×10 ^−5^	0.03
*MIR7-2*	chr15:89155055–89155165	0	10.86	5.0×10 ^−5^	0.03
*ODAM*	chr4:71062243–71070293	0.77	0	5.0×10 ^−5^	0.03
*ONECUT1*	chr15:53049352–53082209	0	2.78	5.0×10 ^−5^	0.03
*PADI3*	chr1:17575592–17610727	0.77	0	5.0×10 ^−5^	0.03
*PITX2*	chr4:111538579–111563279	16.01	146.53	5.0×10 ^−5^	0.03
*PTH*	chr11:13513600–13517567	0	1.71	5.0×10 ^−5^	0.03
*RDH10*	chr8:74206836–74237520	41.71	4.72	5.0×\10 ^−5^	0.03
*RPS6KA2*	chr6:166822853–167275771	37.64	6.46	5.0×10 ^−5^	0.03
*SEC14L3*	chr22:30855215–30868034	1.29	0	5.0×10 ^−5^	0.03
*SEMA7A*	chr15:74701629–74726299	1.34	83.58	5.0×10 ^−5^	0.03
*SLC10A4*	chr4:48485359–48491541	6.17	109.53	5.0 × 10 ^−5^	0.03
*SMARCD3*	chr7:150936058–150974231	37.70	169.90	5.0×10 ^−5^	0.03
*SPRR2F*	chr1:153084612–153085989	0	0.60	5.0×10 ^−5^	0.03
*SSC5D*	chr19:55999869–56030466	6.20	81.08	5.0×10 ^−5^	0.03
*TFF1*	chr21:43782390–43786644	2.09	0	5.0×10 ^−5^	0.03
*UNCX*	chr7:1272653–1276613	0	2.56	5.0×10 ^−5^	0.03

a FPKM: fragments per kilo base of exon per million fragments mapped.

## Discussion

Genome-scale DNA methylation screening of PAs was performed to investigate whether DNA methylation was associated with PA invasion and histopathological subtype classification. To our knowledge, this is the first genome-scale DNA methylation analysis of sporadic PAs, demonstrating that epigenetic modification of key gene substrates may in part account for functional differentiation of PAs, and that DNA methylation analysis of key candidate genes may potentially be used to complement PA current histopathology subtype classification systems.

Tumor invasion has been observed to occur in up to 85% of surgically-resected PAs, based on microscopic analysis of dural samples [30]. Consequently, tumor invasion is perhaps the greatest barrier to achieving adequate tumor control in PAs, as complete surgical resection of noninvasive PAs is typically achieved in 70–95% of noninvasive PA cases, compared with only 20–40% of invasive PAs [31]. In our study, no significant global differences in CpG methylation were identified between invasive and noninvasive PAs. Although CpG island hypermethylation of a series of well-characterized cell cycle regulation genes, including retinoblastoma 1 (*RB1*) [32], *CDKN2A* [33], [34], *and GADD45G* [35] have been linked to gene repression and negative regulated cell growth in PA, these genes were not demonstrated to contribute to the invasive PA phenotype in our study. Importantly, no significant differences in *CDKN2A* methylation were observed between noninvasive and invasive NFAs in our study, a finding which is consistent with prior observations [7], and may also suggest that *CDKN2A* inactivation is alternatively related to PA subtype and size [36]. The current findings suggest that genome-scale DNA methylation assessment may be utilized to identify candidate genes involved in PA invasion that would otherwise possibly be concealed in targeted gene studies. In our study, a secondary analysis showed that the angiogenic gene *SLIT3* [37] and oncogene *FLT1* [38] may be two candidate biomarkers for invasive PAs, if validated in future studies with a larger PA sample size. Meanwhile, although a previous study showed that promoter hypermethylation of *CDH13* and *CDH1* was detected in PAs but not in normal pituitary tissue, and promoter hypermethylation of *CDH13* was observed more frequently in invasive PAs [11], there was no significant global methylation difference between invasive and noninvasive PAs in our study. We did, however, find gene body hypermethylation in *CDH7*, a type 2 classical cadherin from the cadherin superfamily, when comparing noninvasive to invasive NFAs (FDR  =  0.01). Further validation in a larger PA sample population is nevertheless warranted to identify and confirm genomic and epigenomic pathways involved in tumor invasion. Meanwhile, other epigenetic mechanisms (i.e. histone acetylation) should be explored in an effort to explain the character of PA invasion and prioritize genes associated with this clinically significant phenotype.

The anterior pituitary gland (adenohypophysis) secretes six known hormones that regulate homeostasis, including adrenocorticotropic hormone (ACTH), growth hormone (GH), prolactin, thyroid-stimulating hormone (TSH), follicle-stimulating hormone (FSH) and luteinizing hormone (LH) [39]. Clinically nonfunctioning PAs are not able to synthesize and/or secrete functional hormones, although in a majority of cases they nevertheless demonstrate positive immunostaining for LH, FSH, and/or the alpha-subunit [40]. PAs are monoclonal in origin and typically benign tumors, suggesting they arise from expansion of single precursor cells that possess a unique proliferative advantage [41]. These monoclonal adenomas therefore secrete specific hormones reflective of their differentiated cell of origin. In the current study, hierarchical clustering analysis readily separated somatotroph (GH) adenomas from nonfunctional adenomas, suggesting a potential role of DNA methylation in the differentiation and/or functional regulation of these tumors. Furthermore, an unexpected and interesting finding in the current study was that the functional corticotroph adenoma causing Cushing's disease and the silent corticotroph adenoma clustered to the same hierarchical group (group 2).

The exact mechanisms by which nonfunctioning PAs retain immunostaining but fail to produce clinically-active hormones remains to be determined. Our genome-scale screening of variability in DNA methylation between functional and nonfunctional PAs suggests that nonfunctional PAs are globally hypermethylated compared to functional ones. This finding is particularly interesting in that the most differentially methylated gene, *KCNAB2,* encodes a potassium ion-channel that has been previously implicated in endocrine function pertaining to insulin secretion [42]. Pituitary cells resemble neurons and muscle fibers in that they also fire action potentials (APs) [43], which are mediated via expression of numerous voltage-gated sodium, potassium, calcium and chloride channels. These APs are accompanied by a rise in intracellular calcium and spontaneous electrical activity that drives intracellular calcium concentrations above the threshold for stimulus-secretion and stimulus-transcription coupling. *KCNAB2* is a subunit of the shaker-related voltage dependent potassium channel, and upon binding to K^+^ channel alpha subunits, contributes to regulation of channel excitability. In addition, *KCNAB2* has been demonstrated as an functional aldo-ketoreductase (*AKR*) [44], [45]. Among the primarily hypermethylated genes in NFAs, we also identified four significant genes *PSEN2* [46], *MDN1* [47], *PCSK6* and *NME9* [48], which were relatively hypormethylated in NFAs compared to FAs (Table 2). Importantly, *PCSK6* has been reported to be a member of the mammalian subtilisin-like proprotein convertase family, which participates in maturation of precursor protein, and is expressed at high levels in the anterior pituitary gland [49]. This link potentially explains the significant DNA methylation differences in *PCSK6* methylation between NFA and FA, and suggests that *PCSK6* may therefore be an important biomarker for distinguishing FA from NFA. Collectively, these data suggest that genome-scale DNA methylation analysis may provide a practical, complementary molecular correlate to standard PA classification schemes. Further analysis in a larger sample size is required to support the validity of these findings.

Additionally, our study demonstrates that the ion-channel activity signal pathway shows a relative hypermethylated profile in nonfunctional PA subjects. In particular, the pivotal gene *KCNMB4* encodes an important subunit of potassium channels and is expressed throughout the brain (especially in the thalamus and the brainstem) [50]. In addition, transcription of the calcium channel gene *CACNA1C* has been reported to undergo epigenetic regulation via DNA methylation [51]. Taken together, these findings suggest that modulation of ion activity pathways may contribute to defective hormone secretion in nonfunctional PAs, and possibly serve as novel candidate biomarkers in PAs.

Finally, in order to investigate the association of DNA methylation and gene expression, total RNA sequencing and differential expression analysis was performed. Although the expression of *KCNAB2* and other genes in ion-channel activity signal pathway was not significantly different between FAs and NFAs, our global correlation analysis showed that DNA hypermethylation was negatively associated with gene expression. The elevated expression of *ODAM* in FAs provides a potentially novel insight to the pathogenesis of epithelial neoplasms, since *ODAM* is a developmental antigen with an essential role in tooth maturation and the pathogenesis of various epithelial neoplasms [52]. Although we explored differences in RNA expression of *KCNAB2* between NFAs and FAs, the result was not significant. A larger sample size would likely be needed to validate this association. Because gene expression is modulated not only by epigenome modification, low levels of gene expression cannot be attributed solely to hypermethylation. Additionally, the intra-tumoral multifocality and heterogeneity of DNA methylation of the PAs may account for this abberant association of DNA methylation and gene expression [53]. There are several limitations to the current study, including a relatively small sample size and nonuniform representation of functional to nonfunctional PAs and various histopathological PA subtypes. Furthermore, MethyLight validation of only selected candidate genes was performed, mandating further validation in a larger sample size and using another technique. The mechanisms by which methylation alterations of selected candidate genes result in observed invasion or hormonal phenotypes were also not studied, and remain beyond the scope of the current study. Finally, the current study is limited in that no true control samples (normal pituitary gland) were included in the analysis. Nevertheless, the current study suggests that DNA methylation analysis can be used to provide valuable insight into the phenotype of histopathological subtype in PAs, and if validated may complement current pathological classification systems. The current study is the first genome-wide DNA methylation analysis in PAs, and can be used to appropriately design and power future studies with a larger sample size in order to validate many of the preliminary findings from our study.

In conclusion, genome-scale DNA methylation profiling and RNA sequencing of PAs identified DNA methylation variations in candidate genes associated with functional subtype (both globally and in selected genes) and possibly invasion. Hierarchical clustering analysis showed PA clustering according to functional status and immunohistochemical subtype, suggesting that DNA methylation analysis may possibly provide a clinically useful and complementary molecular correlate to standard PA classification. Differential methylation of cell motility related genes (such as *FLT1* and *SLIT3*) require further validation prior to being considered candidate biomarkers for PA invasion. DNA hypermethylation of *KCNAB2* and enrichment of DNA methylation in ion-channel activity signal pathways may be associated with the endocrine-inactive status of nonfunctional PAs.

## Supporting Information

File S1 **Analysis strategies.** Figure A, Filtering strategy for Illumina HM450 Probes. SNP10: SNPs within 10 bp from CpG site; SNP15: SNPs within 15 bp from the CpG site lying entirely within a repeat region; ChrX: chromosome X. Figure B, Additional filters were used in the secondary analysis of the invasive phenotype of NFAs. A total of 884 probes were enrolled in further analysis, of which 355 were enhancer (Enh)- associated, 20 were promoter (Pro)- associated, and 4 were both (Bot) promoter- and enhancer-associated, and we identified 34 significant (Sig) CpGs that were independently associated with enhancers and were hypomethylated in invasive NFAs compared to noninvasive NFAs.
(TIF)

File S2 **The distribution of probes on the Illumina HM450 DNA methylation platform.** Figure A, The distribution of 383,718 HM450 probes stratified by CpG density (in and out of CpG islands). Figure B, The distribution of differentially methylated CpGs across different gene-related and intergenic regions.
(TIF)

File S3 **Hierarchical Clustering of DNA methylation in 24 PA cases.** The HM450 probes with the highest 1% of standard deviation across the set of 383,718 global HM450 probes were used.
(TIF)

File S4 **Hierarchical Clustering of DNA methylation in 24 PA cases.** The HM450 probes with the highest 5% of standard deviation across the set of 383,718 global HM450 probes were used.
(TIF)

File S5 **Hierarchical clustering of DNA methylation levels within the** ***KCNAB2*** **gene locus.** Beta values with 50% of the highest standard deviation were used. Pro-a: promoter associated. Compared with FAs, NFAs showed relative hypermethylation across nearly the whole gene, especially in promoter-associated CpG sets.
(TIF)

File S6 **Enrichment plots of significant gene sets.** The score at the peak of the plot is the enrichment score for the gene set. “0” represents NFAs, and “1” represents FAs.
(TIF)

File S7 **Heat map of genes in the ion-channel activity signal gene set.** DNA methylation values are represented as colors, with red representing DNA hypermethylation and blue representing DNA hypomethylation.
(TIF)

File S8 **Leading edge analysis of the four overlapping significant gene sets.** Figure A, Darker color represents greater overlap between the subsets. The gene set ion-channel activity signal pathway had a lot of overlap with the substrate specific channel activity signal. Figure B, *KCNMB4* and *CACNA1C* were enriched in all of the four gene sets. DNA methylation values were represented as colors, where the range of colors (red, pink, light blue and dark blue) shown the range of methylation values (high, moderate, low and lowest).
(TIF)

Table S1 **Top 17 significant genes with most differentially-methylated enhancers between invasive and noninvasive NFAs.**
(XLSX)

Table S2 **Hypermethylation genes in NFAs, which are consistent with results of Illumina Infinium Methylation 27K arrays.**
(XLSX)

Table S3 **Significant gene set enrichment in NFAs.** Four significant pathways were listed in the table, and ion-channel activity pathway showed high enrichment score with the lowest FDR value.
(XLSX)

Table S4 **Gene subset of ion-channel activity signal pathway.** The majority of genes were associated with potassium channels.
(XLSX)

Table S5 **Full names of genes mentioned in the text.**
(XLSX)

## References

[1] Ezzat S, Asa SL, Couldwell WT, Barr CE, Dodge WE, et al (2004) The prevalence of pituitary adenomas: a systematic review. Cancer 101: 613–619. 15274075 10.1002/cncr.20412

[2] Nammour GM, Ybarra J, Naheedy MH, Romeo JH, Aron DC (1997) Incidental pituitary macroadenoma: a population-based study. Am J Med Sci 314: 287–291. 9365329 10.1097/00000441-199711000-00003

[3] Zada G, Woodmansee WW, Ramkissoon S, Amadio J, Nose V, et al (2011) Atypical pituitary adenomas: incidence, clinical characteristics, and implications. J Neurosurg 114: 336–344. 20868211 10.3171/2010.8.JNS10290

[4] Knosp E, Steiner E, Kitz K, Matula C (1993) Pituitary adenomas with invasion of the cavernous sinus space: a magnetic resonance imaging classification compared with surgical findings. Neurosurgery 33: 610–617 discussion 617–618. 8232800 10.1227/00006123-199310000-00008

[5] Chandrasekharappa SC, Guru SC, Manickam P, Olufemi SE, Collins FS, et al (1997) Positional cloning of the gene for multiple endocrine neoplasia-type 1. Science 276: 404–407. 9103196 10.1126/science.276.5311.404

[6] Dudley KJ, Revill K, Clayton RN, Farrell WE (2009) Pituitary tumours: all silent on the epigenetics front. J Mol Endocrinol 42: 461–468. 19208779 10.1677/JME-09-0009

[7] Simpson DJ, Bicknell JE, McNicol AM, Clayton RN, Farrell WE (1999) Hypermethylation of the p16/CDKN2A/MTSI gene and loss of protein expression is associated with nonfunctional pituitary adenomas but not somatotrophinomas. Genes Chromosomes Cancer 24: 328–336. 10092131

[8] Woloschak M, Yu A, Post KD (1997) Frequent inactivation of the p16 gene in human pituitary tumors by gene methylation. Mol Carcinog 19: 221–224. 9290697 10.1002/(sici)1098-2744(199708)19:4<221::aid-mc1>3.0.co;2-f

[9] Bahar A, Bicknell JE, Simpson DJ, Clayton RN, Farrell WE (2004) Loss of expression of the growth inhibitory gene GADD45gamma, in human pituitary adenomas, is associated with CpG island methylation. Oncogene 23: 936–944. 14647444 10.1038/sj.onc.1207193

[10] Farrell WE (2006) A novel apoptosis gene identified in the pituitary gland. Neuroendocrinology 84: 217–221. 17135715 10.1159/000097486

[11] Qian ZR, Sano T, Yoshimoto K, Asa SL, Yamada S, et al (2007) Tumor-specific downregulation and methylation of the CDH13 (H-cadherin) and CDH1 (E-cadherin) genes correlate with aggressiveness of human pituitary adenomas. Mod Pathol 20: 1269–1277. 17873891 10.1038/modpathol.3800965

[12] Yuan Y, Qian ZR, Sano T, Asa SL, Yamada S, et al (2008) Reduction of GSTP1 expression by DNA methylation correlates with clinicopathological features in pituitary adenomas. Mod Pathol 21: 856–865. 18425080 10.1038/modpathol.2008.60

[13] Duong CV, Emes RD, Wessely F, Yacqub-Usman K, Clayton RN, et al (2012) Quantitative, genome-wide analysis of the DNA methylome in sporadic pituitary adenomas. Endocr Relat Cancer 19: 805–816. 23045325 10.1530/ERC-12-0251

[14] Pease M, Ling C, Mack WJ, Wang K, Zada G (2013) The role of epigenetic modification in tumorigenesis and progression of pituitary adenomas: a systematic review of the literature. PLoS One 8: e82619. 24367530 10.1371/journal.pone.0082619 PMC3867353

[15] Triche TJ Jr, Weisenberger DJ, Van Den Berg D, Laird PW, Siegmund KD (2013) Low-level processing of Illumina Infinium DNA Methylation BeadArrays. Nucleic Acids Res 41: e90. 23476028 10.1093/nar/gkt090 PMC3627582

[16] Saeed AI, Bhagabati NK, Braisted JC, Liang W, Sharov V, et al (2006) TM4 microarray software suite. Methods Enzymol 411: 134–193. 16939790 10.1016/S0076-6879(06)11009-5

[17] Weisenberger DJ, Siegmund KD, Campan M, Young J, Long TI, et al (2006) CpG island methylator phenotype underlies sporadic microsatellite instability and is tightly associated with BRAF mutation in colorectal cancer. Nat Genet 38: 787–793. 16804544 10.1038/ng1834

[18] Weisenberger DJ, Campan M, Long TI, Kim M, Woods C, et al (2005) Analysis of repetitive element DNA methylation by MethyLight. Nucleic Acids Res 33: 6823–6836. 16326863 10.1093/nar/gki987 PMC1301596

[19] Subramanian A, Tamayo P, Mootha VK, Mukherjee S, Ebert BL, et al (2005) Gene set enrichment analysis: a knowledge-based approach for interpreting genome-wide expression profiles. Proc Natl Acad Sci U S A 102: 15545–15550. 16199517 10.1073/pnas.0506580102 PMC1239896

[20] Ebert MP, Tanzer M, Balluff B, Burgermeister E, Kretzschmar AK, et al (2012) TFAP2E-DKK4 and chemoresistance in colorectal cancer. N Engl J Med 366: 44–53. 22216841 10.1056/NEJMoa1009473

[21] Payne SR, Serth J, Schostak M, Kamradt J, Strauss A, et al (2009) DNA methylation biomarkers of prostate cancer: confirmation of candidates and evidence urine is the most sensitive body fluid for non-invasive detection. Prostate 69: 1257–1269. 19459176 10.1002/pros.20967

[22] Chambers TJ, Giles A, Brabant G, Davis JR (2013) Wnt signalling in pituitary development and tumorigenesis. Endocr Relat Cancer 20: R101–111. 23689382 10.1530/ERC-13-0005

[23] McCormack T, McCormack K, Nadal MS, Vieira E, Ozaita A, et al (1999) The effects of Shaker beta-subunits on the human lymphocyte K+ channel Kv1.3. J Biol Chem 274: 20123–20126. 10400624 10.1074/jbc.274.29.20123

[24] Yu FH, Yarov-Yarovoy V, Gutman GA, Catterall WA (2005) Overview of molecular relationships in the voltage-gated ion channel superfamily. Pharmacol Rev 57: 387–395. 16382097 10.1124/pr.57.4.13

[25] Herman JG, Merlo A, Mao L, Lapidus RG, Issa JP, et al (1995) Inactivation of the CDKN2/p16/MTS1 gene is frequently associated with aberrant DNA methylation in all common human cancers. Cancer Res 55: 4525–4530. 7553621

[26] Baylin SB, Herman JG (2000) DNA hypermethylation in tumorigenesis: epigenetics joins genetics. Trends Genet 16: 168–174. 10729832 10.1016/s0168-9525(99)01971-x

[27] Garcia L, Fahmi M, Prevarskaya N, Dufy B, Sartor P (1997) Modulation of voltage-dependent Ca2+ conductance by changing Cl- concentration in rat lactotrophs. Am J Physiol 272: C1178–1185. 9142842 10.1152/ajpcell.1997.272.4.C1178

[28] Gonzalez-Iglesias AE, Jiang Y, Tomic M, Kretschmannova K, Andric SA, et al (2006) Dependence of electrical activity and calcium influx-controlled prolactin release on adenylyl cyclase signaling pathway in pituitary lactotrophs. Mol Endocrinol 20: 2231–2246. 16645040 10.1210/me.2005-0363

[29] Jones PA (2012) Functions of DNA methylation: islands, start sites, gene bodies and beyond. Nat Rev Genet 13: 484–492. 22641018 10.1038/nrg3230

[30] Selman WR, Laws ER Jr, Scheithauer BW, Carpenter SM (1986) The occurrence of dural invasion in pituitary adenomas. J Neurosurg 64: 402–407. 3950720 10.3171/jns.1986.64.3.0402

[31] Zada G, Kelly DF, Cohan P, Wang C, Swerdloff R (2003) Endonasal transsphenoidal approach for pituitary adenomas and other sellar lesions: an assessment of efficacy, safety, and patient impressions. J Neurosurg 98: 350–358. 12593622 10.3171/jns.2003.98.2.0350

[32] Ogino A, Yoshino A, Katayama Y, Watanabe T, Ota T, et al (2005) The p15(INK4b)/p16(INK4a)/RB1 pathway is frequently deregulated in human pituitary adenomas. J Neuropathol Exp Neurol 64: 398–403. 15892297 10.1093/jnen/64.5.398

[33] Farrell WE, Simpson DJ, Frost SJ, Clayton RN (1999) Methylation mechanisms in pituitary tumorigenesis. Endocr Relat Cancer 6: 437–447. 10730899 10.1677/erc.0.0060437

[34] Jaffrain-Rea ML, Ferretti E, Toniato E, Cannita K, Santoro A, et al (1999) p16 (INK4a, MTS-1) gene polymorphism and methylation status in human pituitary tumours. Clin Endocrinol (Oxf) 51: 317–325. 10469011 10.1046/j.1365-2265.1999.00774.x

[35] Mezzomo LC, Gonzales PH, Pesce FG, Kretzmann Filho N, Ferreira NP, et al (2012) Expression of cell growth negative regulators MEG3 and GADD45gamma is lost in most sporadic human pituitary adenomas. Pituitary 15: 420–427. 21850407 10.1007/s11102-011-0340-1

[36] Seemann N, Kuhn D, Wrocklage C, Keyvani K, Hackl W, et al (2001) CDKN2A/p16 inactivation is related to pituitary adenoma type and size. J Pathol 193: 491–497. 11276008 10.1002/path.833

[37] Zhang B, Dietrich UM, Geng JG, Bicknell R, Esko JD, et al (2009) Repulsive axon guidance molecule Slit3 is a novel angiogenic factor. Blood 114: 4300–4309. 19741192 10.1182/blood-2008-12-193326 PMC2774558

[38] Kaplan RN, Riba RD, Zacharoulis S, Bramley AH, Vincent L, et al (2005) VEGFR1-positive haematopoietic bone marrow progenitors initiate the pre-metastatic niche. Nature 438: 820–827. 16341007 10.1038/nature04186 PMC2945882

[39] Melmed S (2003) Mechanisms for pituitary tumorigenesis: the plastic pituitary. J Clin Invest 112: 1603–1618. 14660734 10.1172/JCI20401 PMC281651

[40] Black PM, Hsu DW, Klibanski A, Kliman B, Jameson JL, et al (1987) Hormone production in clinically nonfunctioning pituitary adenomas. J Neurosurg 66: 244–250. 3543255 10.3171/jns.1987.66.2.0244

[41] Herman V, Fagin J, Gonsky R, Kovacs K, Melmed S (1990) Clonal origin of pituitary adenomas. J Clin Endocrinol Metab 71: 1427–1433. 1977759 10.1210/jcem-71-6-1427

[42] Dai XQ, Manning Fox JE, Chikvashvili D, Casimir M, Plummer G, et al (2012) The voltage-dependent potassium channel subunit Kv2.1 regulates insulin secretion from rodent and human islets independently of its electrical function. Diabetologia 55: 1709–1720. 22411134 10.1007/s00125-012-2512-6

[43] Kidokoro Y (1975) Spontaneous calcium action potentials in a clonal pituitary cell line and their relationship to prolactin secretion. Nature 258: 741–742. 813152 10.1038/258741a0

[44] McCormack K, Connor JX, Zhou L, Ho LL, Ganetzky B, et al (2002) Genetic analysis of the mammalian K+ channel beta subunit Kvbeta 2 (Kcnab2). J Biol Chem 277: 13219–13228. 11825900 10.1074/jbc.M111465200

[45] Weng J, Cao Y, Moss N, Zhou M (2006) Modulation of voltage-dependent Shaker family potassium channels by an aldo-keto reductase. J Biol Chem 281: 15194–15200. 16569641 10.1074/jbc.M513809200 PMC2862575

[46] Andreoli V, Trecroci F, La Russa A, Di Palma G, Quattrone A, et al (2008) Gene symbol: PSEN2. Disease: Alzheimer disease. Hum Genet 124: 304. 18846634

[47] Bassler J, Kallas M, Pertschy B, Ulbrich C, Thoms M, et al (2010) The AAA-ATPase Rea1 drives removal of biogenesis factors during multiple stages of 60S ribosome assembly. Mol Cell 38: 712–721. 20542003 10.1016/j.molcel.2010.05.024 PMC3372891

[48] Desvignes T, Pontarotti P, Fauvel C, Bobe J (2009) Nme protein family evolutionary history, a vertebrate perspective. BMC Evol Biol 9: 256. 19852809 10.1186/1471-2148-9-256 PMC2777172

[49] Dong W, Marcinkiewicz M, Vieau D, Chretien M, Seidah NG, et al (1995) Distinct mRNA expression of the highly homologous convertases PC5 and PACE4 in the rat brain and pituitary. J Neurosci 15: 1778–1796. 7891135 10.1523/JNEUROSCI.15-03-01778.1995 PMC6578130

[50] Piwonska M, Wilczek E, Szewczyk A, Wilczynski GM (2008) Differential distribution of Ca2+-activated potassium channel beta4 subunit in rat brain: immunolocalization in neuronal mitochondria. Neuroscience 153: 446–460. 18359571 10.1016/j.neuroscience.2008.01.050

[51] Nishioka M, Shimada T, Bundo M, Ukai W, Hashimoto E, et al (2013) Neuronal cell-type specific DNA methylation patterns of the Cacna1c gene. Int J Dev Neurosci 31: 89–95. 23183239 10.1016/j.ijdevneu.2012.11.007

[52] Kestler DP, Foster JS, Macy SD, Murphy CL, Weiss DT, et al (2008) Expression of odontogenic ameloblast-associated protein (ODAM) in dental and other epithelial neoplasms. Mol Med 14: 318–326. 18472969 10.2119/2008-00010.Kestler PMC2323332

[53] Kekeeva TV, Popova OP, Shegai PV, Alekseev B, Adnreeva I, et al (2007) [Abberant methylation of p16, HIC1, N33 and GSTP1 genes in tumor epitelium and tumor-associated stromal cells of prostate cancer]. Mol Biol (Mosk) 41: 79–85. 17380894

