# Supplementary figures and images for: A Pilot Genome-Scale Profiling of DNA Methylation in Sporadic Pituitary Macroadenomas: Association with Tumor Invasion and Histopathological Subtype
Source: PLoS One. 2014 Apr 29;9(4):e96178. doi: 10.1371/journal.pone.0096178 (PMC4004564; doi:10.1371/journal.pone.0096178)

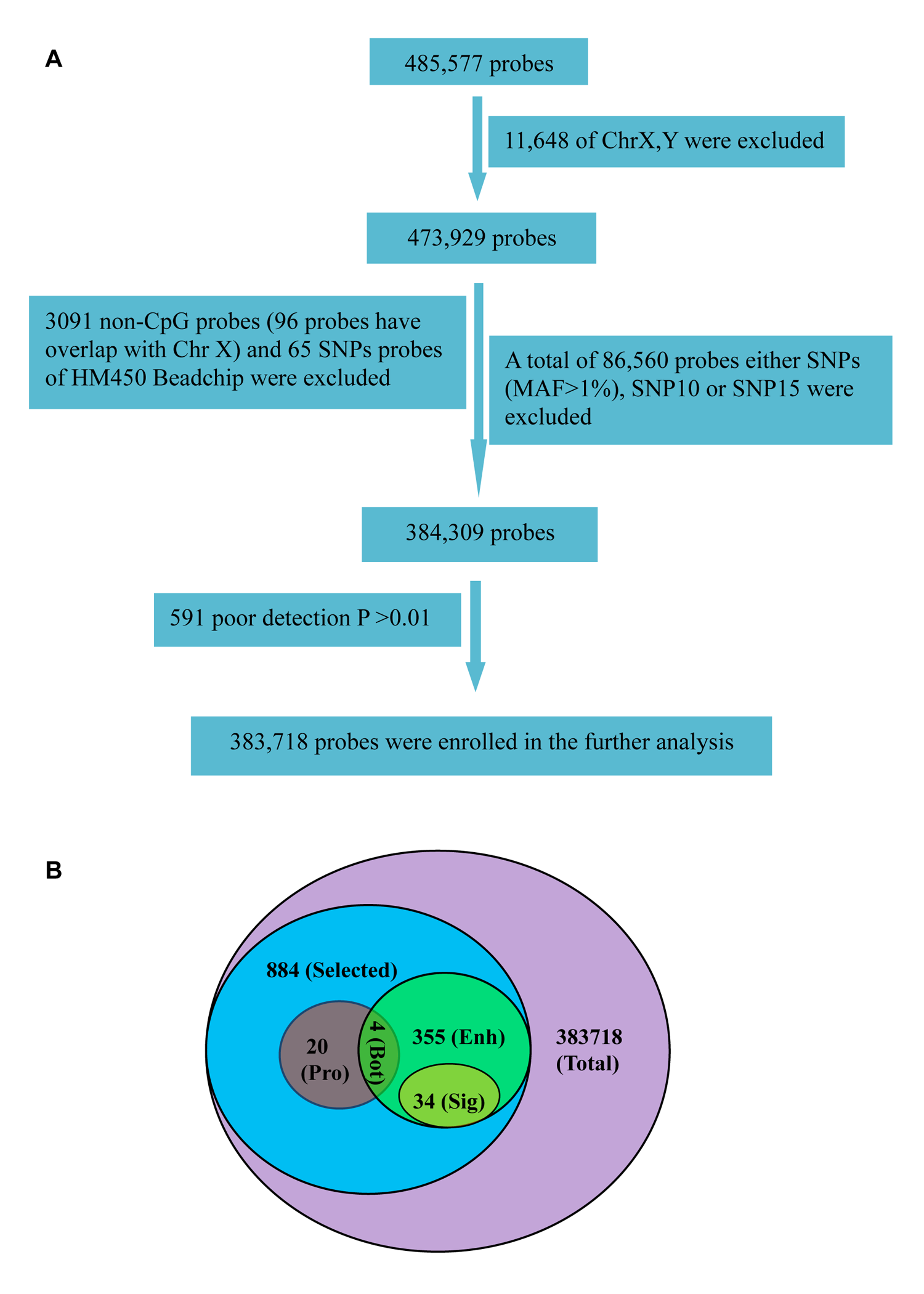

Supplement: File S1 — Analysis strategies. Figure A, Filtering strategy for Illumina HM450 Probes. SNP10: SNPs within 10 bp from CpG site; SNP15: SNPs within 15 bp from the CpG site lying entirely within a repeat region; ChrX: chromosome X. Figure B, Additional filters were used in the secondary analysis of the invasive phenotype of NFAs. A total of 884 probes were enrolled in further analysis, of which 355 were enhancer (Enh)- associated, 20 were promoter (Pro)- associated, and 4 were both (Bot) promoter- and enhancer-associated, and we identified 34 significant (Sig) CpGs that were independently associated with enhancers and were hypomethylated in invasive NFAs compared to noninvasive NFAs. (TIF) [file pone.0096178.s001.tif]

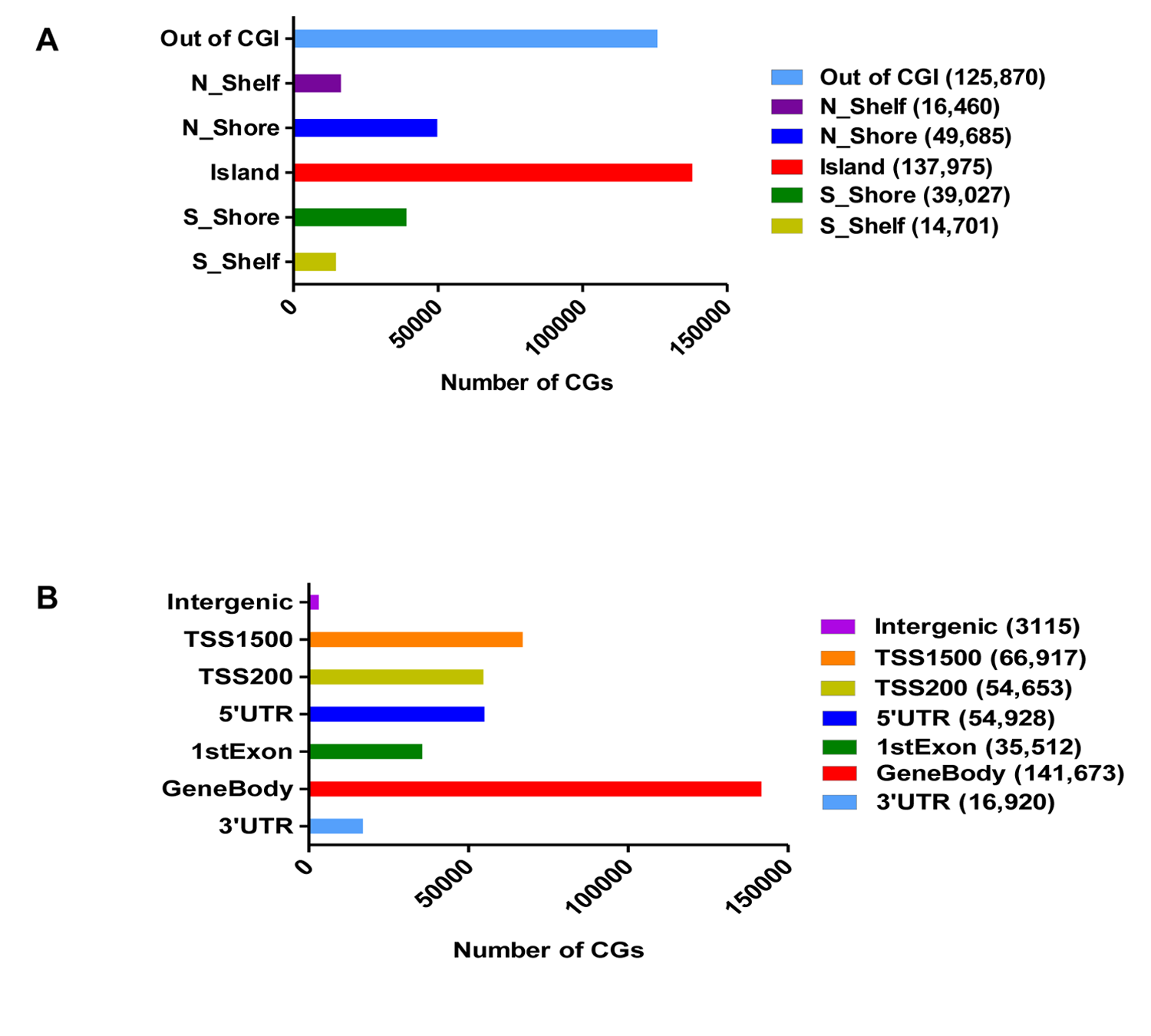

Supplement: File S2 — The distribution of probes on the Illumina HM450 DNA methylation platform. Figure A, The distribution of 383,718 HM450 probes stratified by CpG density (in and out of CpG islands). Figure B, The distribution of differentially methylated CpGs across different gene-related and intergenic regions. (TIF) [file pone.0096178.s002.tif]

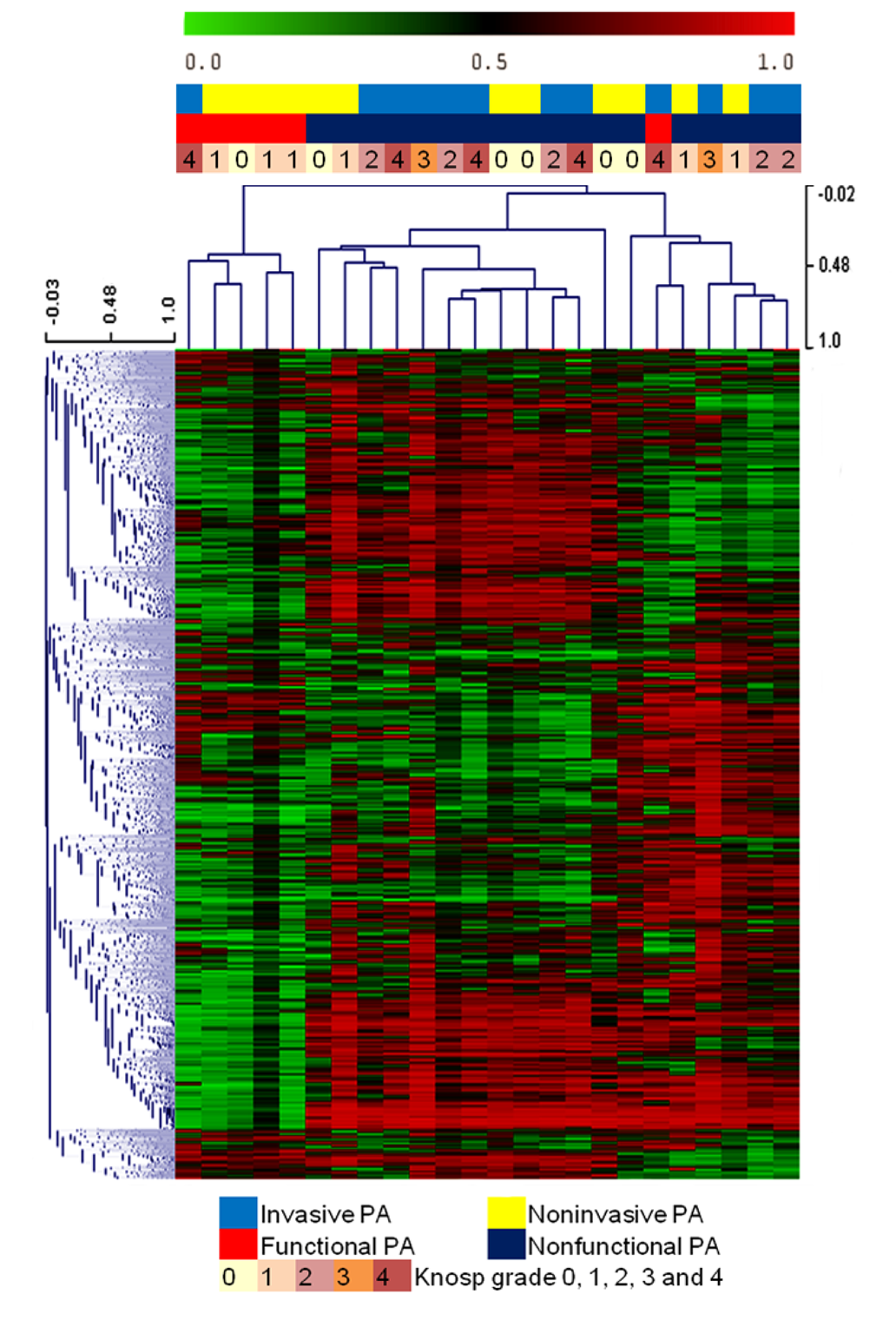

Supplement: File S3 — Hierarchical Clustering of DNA methylation in 24 PA cases. The HM450 probes with the highest 1% of standard deviation across the set of 383,718 global HM450 probes were used. (TIF) [file pone.0096178.s003.tif]

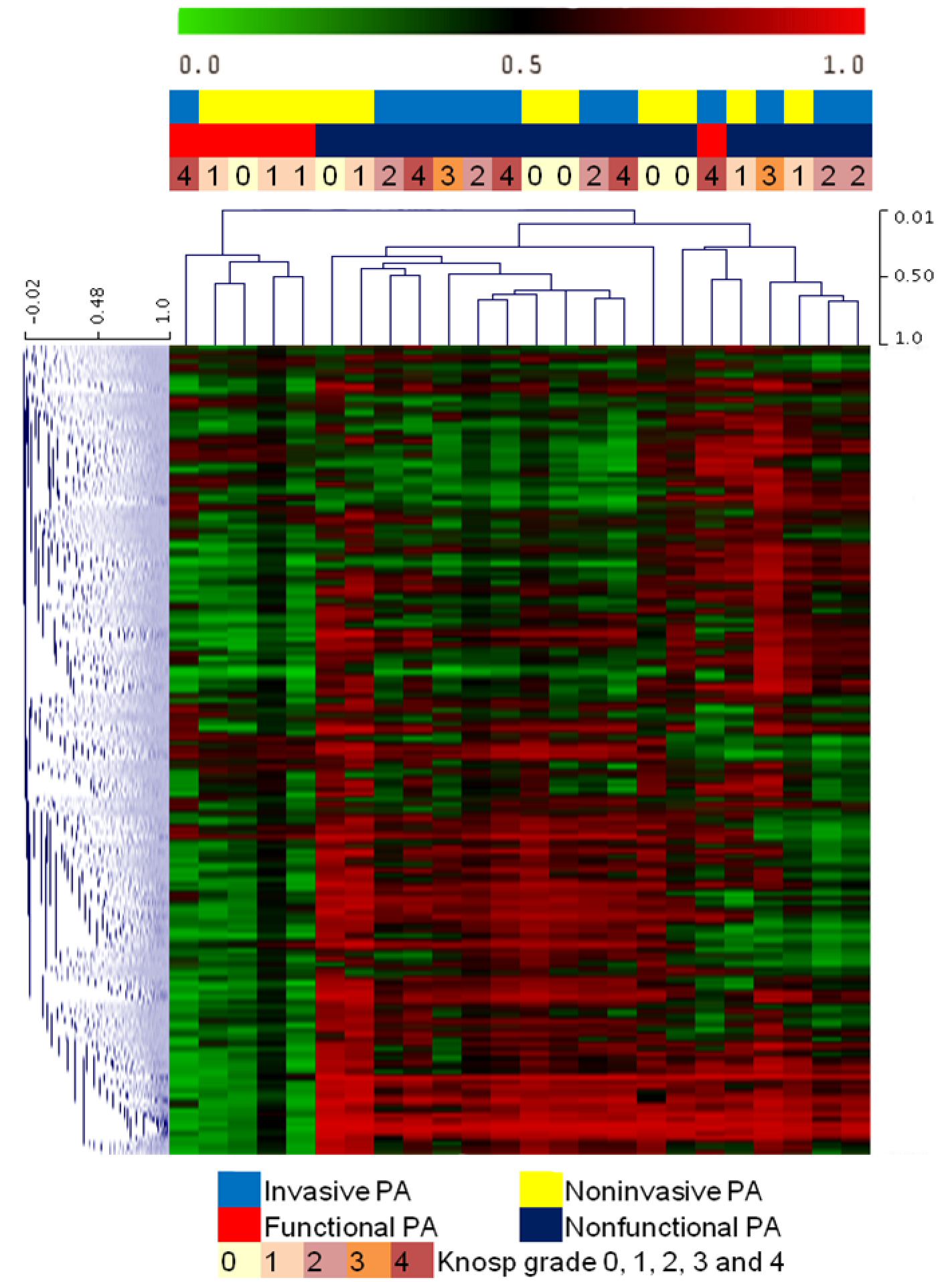

Supplement: File S4 — Hierarchical Clustering of DNA methylation in 24 PA cases. The HM450 probes with the highest 5% of standard deviation across the set of 383,718 global HM450 probes were used. (TIF) [file pone.0096178.s004.tif]

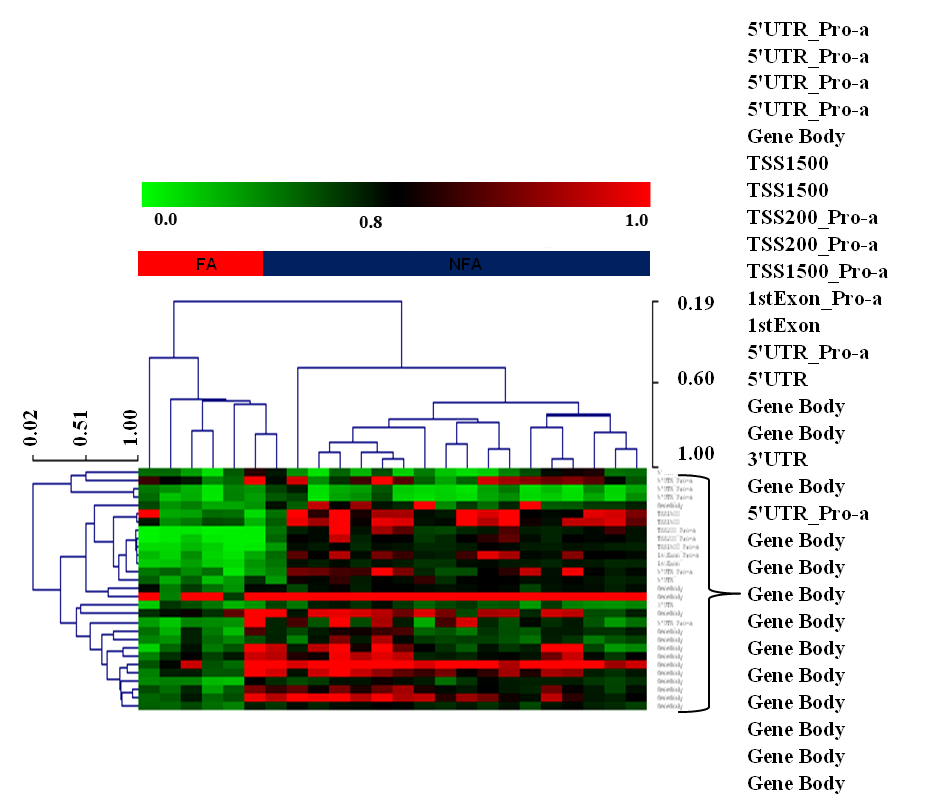

Supplement: File S5 — Hierarchical clustering of DNA methylation levels within the KCNAB2 gene locus. Beta values with 50% of the highest standard deviation were used. Pro-a: promoter associated. Compared with FAs, NFAs showed relative hypermethylation across nearly the whole gene, especially in promoter-associated CpG sets. (TIF) [file pone.0096178.s005.tif]

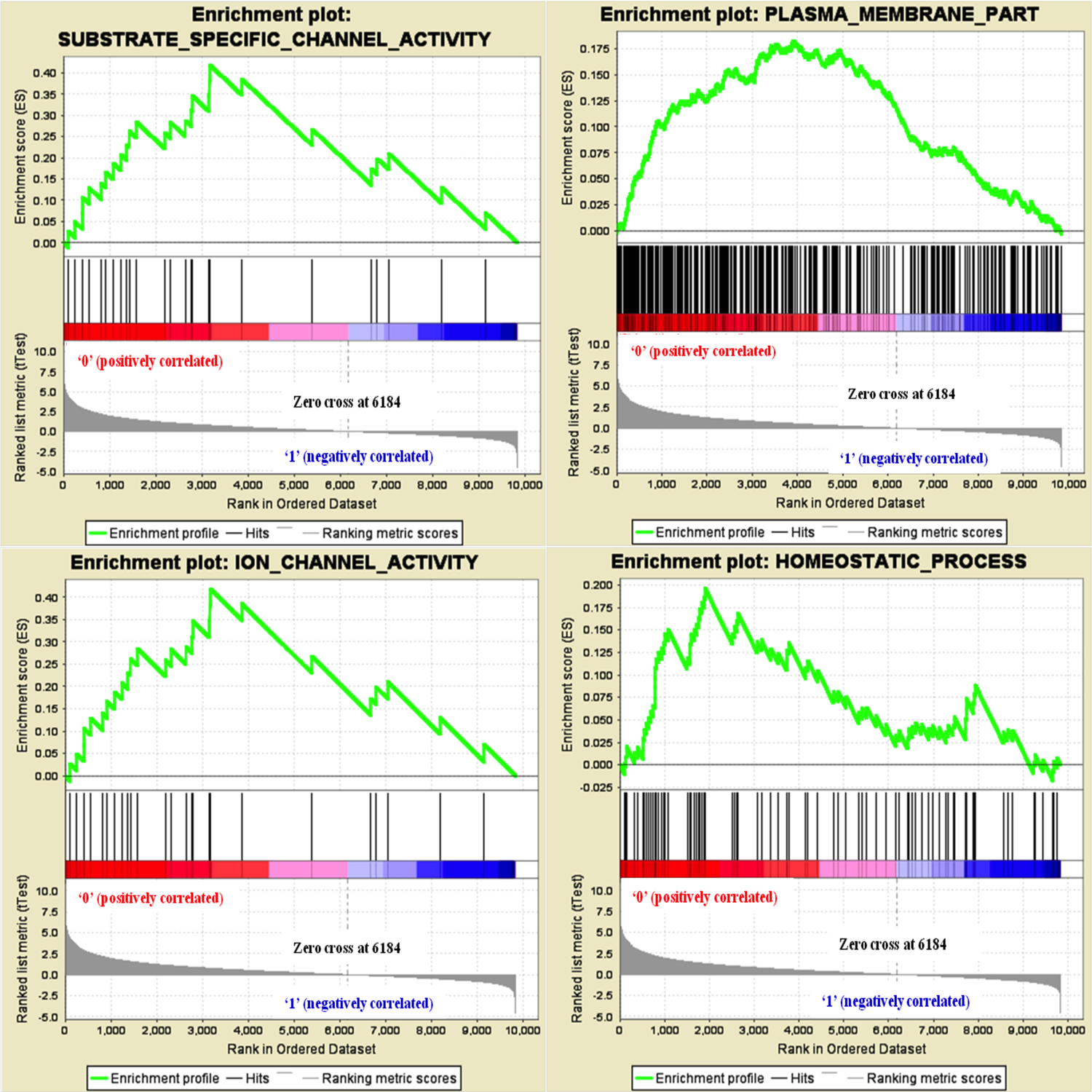

Supplement: File S6 — Enrichment plots of significant gene sets. The score at the peak of the plot is the enrichment score for the gene set. “0” represents NFAs, and “1” represents FAs. (TIF) [file pone.0096178.s006.tif]

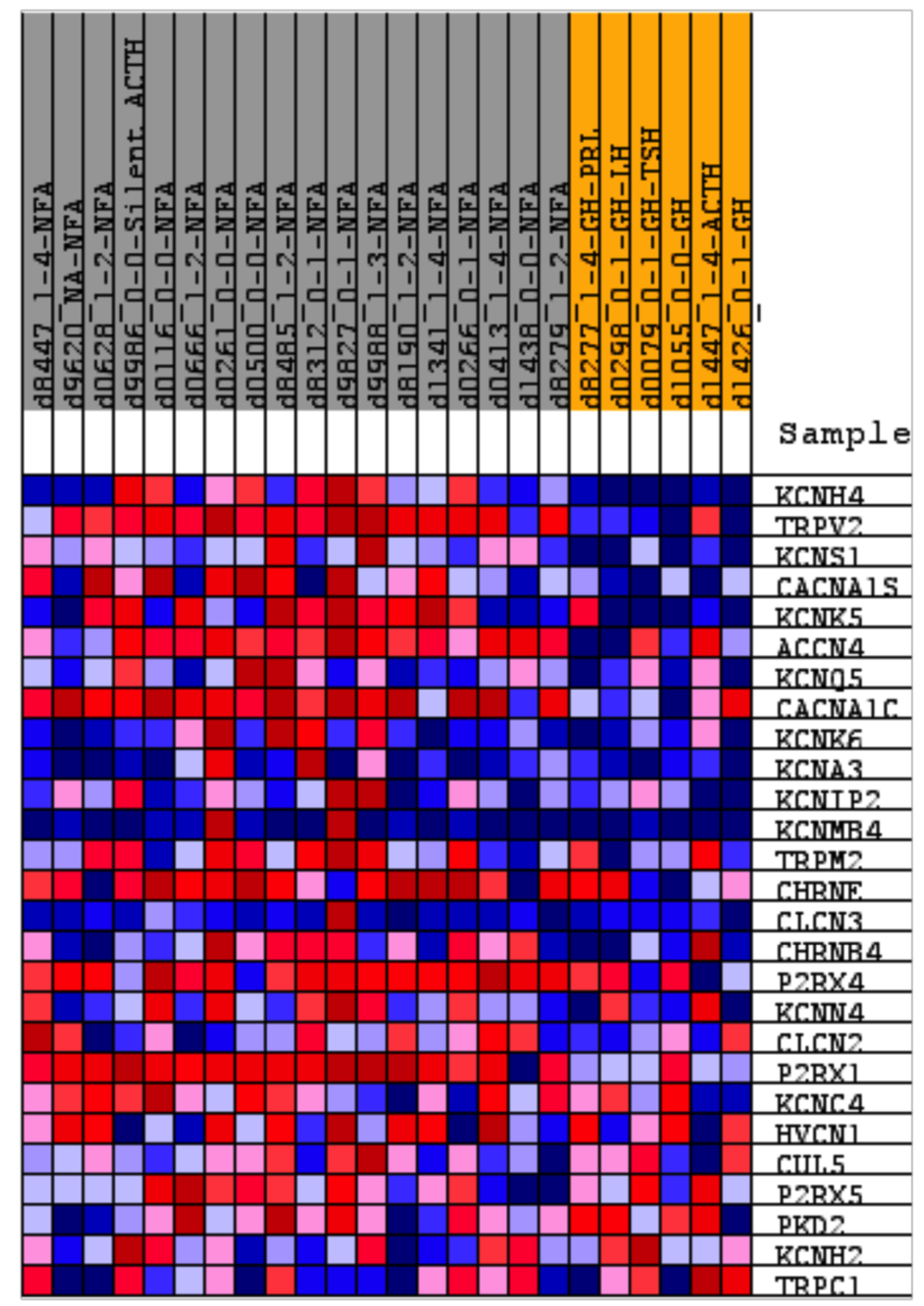

Supplement: File S7 — Heat map of genes in the ion-channel activity signal gene set. DNA methylation values are represented as colors, with red representing DNA hypermethylation and blue representing DNA hypomethylation. (TIF) [file pone.0096178.s007.tif]

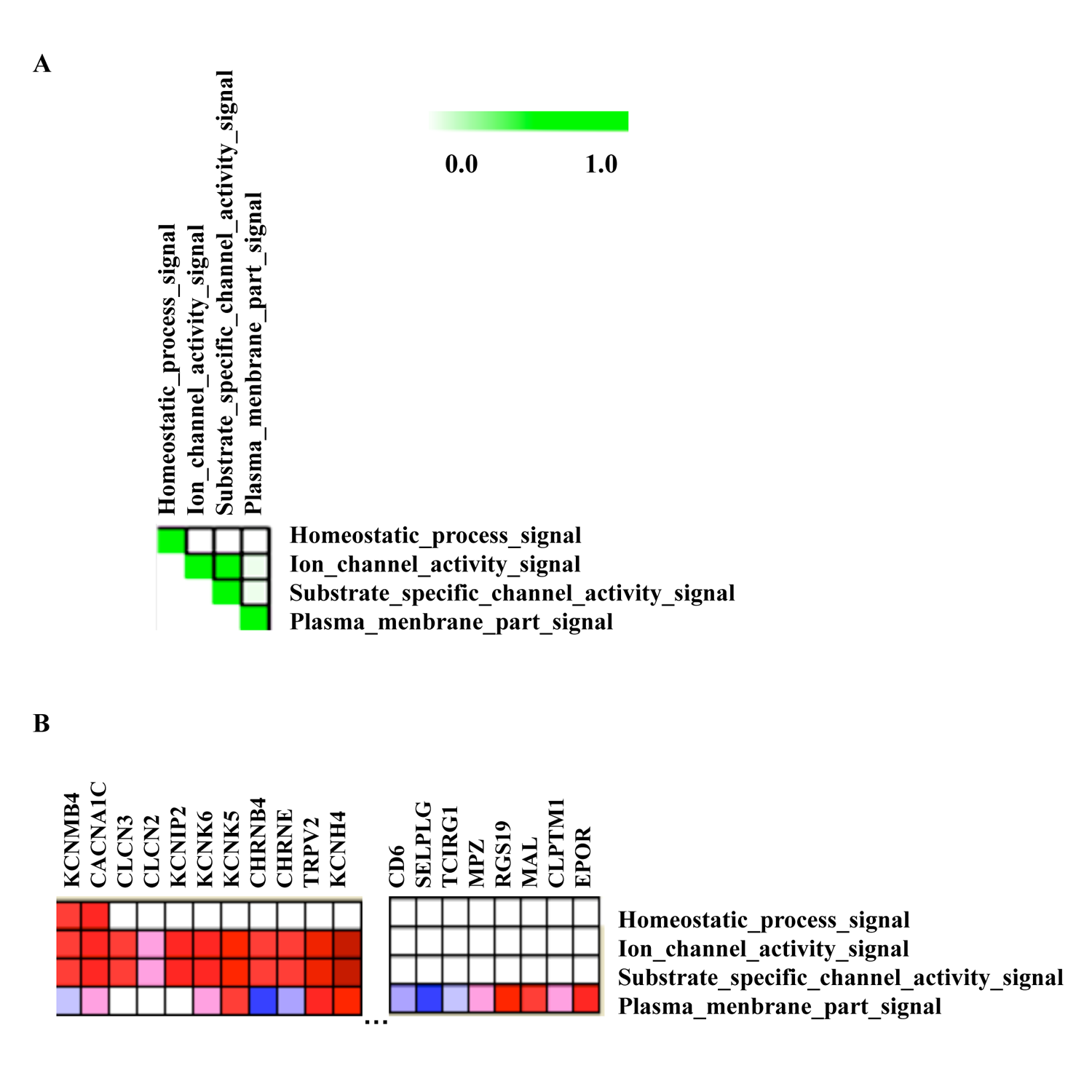

Supplement: File S8 — Leading edge analysis of the four overlapping significant gene sets. Figure A, Darker color represents greater overlap between the subsets. The gene set ion-channel activity signal pathway had a lot of overlap with the substrate specific channel activity signal. Figure B, KCNMB4 and CACNA1C were enriched in all of the four gene sets. DNA methylation values were represented as colors, where the range of colors (red, pink, light blue and dark blue) shown the range of methylation values (high, moderate, low and lowest). (TIF) [file pone.0096178.s008.tif]
